# Characterization of *Streptococcus pneumoniae* isolates obtained from the middle ear fluid of US children, 2011–2021

**DOI:** 10.3389/fped.2024.1383748

**Published:** 2024-07-15

**Authors:** Lindsay R. Grant, Kevin Apodaca, Lalitagauri Deshpande, John H. Kimbrough, Kyla Hayford, Qi Yan, Rodrigo Mendes, Alejandro Cané, Bradford D. Gessner, Adriano Arguedas

**Affiliations:** ^1^ Vaccines and Antivirals, Pfizer Inc., Collegeville, PA, United States; ^2^ JMI Laboratories, North Liberty, IA, United States

**Keywords:** otitis media, *Streptococcus pneumoniae*, pneumococcal conjugate vaccine, serotype distribution, antimicrobial susceptibility

## Abstract

**Introduction:**

Pneumococcal conjugate vaccines (PCVs), including higher valency vaccines such as PCV20, have the potential to reduce pediatric otitis media. We assessed serotype distribution, potential PCV coverage, and antimicrobial susceptibility of *Streptococcus pneumoniae* isolates cultured from middle ear fluid (MEF) of US children age ≤5 years.

**Methods:**

*S. pneumoniae* isolates identified from US hospitals participating in the SENTRY Antimicrobial Surveillance program from 2011 to 2021 were included. Serotypes were determined by in silico analysis based on Pneumococcal Capsular Typing methodology. The percentage of isolates belonging to serotypes included in PCV13 (serotypes 1, 3, 4, 5, 6A, 6B, 7F, 9V, 14, 18C, 19A, 19F, 23F), PCV15 (PCV13 plus 22F, 33F), and PCV20 (PCV13 plus, 8, 10A, 11A, 12F, 15B, 22F and 33F) was calculated. Antimicrobial susceptibility testing was performed by broth microdilution and interpreted using CLSI criteria. Nonsusceptibility was defined as isolates that were intermediate or resistant to a selected antimicrobial.

**Results:**

Among the 199 *S. pneumoniae* isolates that were identified, 56.8% were from children age <2 years. Six serotypes accounted for around 60% of isolates: 35B (16.6%), 15B (14.6%), 15A (7.5%), 19A (7.5%), 19F (7.5%), and 3 (7.0%). Serotypes included in PCV13, PCV15, and PCV20 accounted for 23.1%, 30.2%, and 54.8% of isolates, respectively. Overall, 45.2% of isolates were penicillin non-susceptible, and 13.6% were MDR, of which 48% were serotype 19A. Seven serotypes (19A, 15A, 15B, 15C, 23A, 33F, and 35B) accounted for the majority of non-susceptible isolates.

**Discussion:**

PCVs, particularly PCV20, may prevent a substantial fraction of *S. pneumoniae* otitis media (OM), including OM due to non-susceptible serotypes. The addition of serotypes 15A, 23A, and 35B would improve coverage against susceptible and non-susceptible pneumococcal OM.

## Introduction

*Streptococcus pneumoniae* is one of the most common bacterial pathogens causing otitis media (OM). In 2000, a 7-valent pneumococcal conjugate vaccine (PCV7) was licensed and incorporated into the pediatric national immunization program (NIP) in the US, which was replaced by the 13-valent pneumococcal conjugate vaccine (PCV13) in 2010 (1). Among US children born between 2017 and 2018, 82.3% had received four or more doses of PCV13 by age 24 months (2). Since the introduction of pneumococcal conjugate vaccines (PCV) into the pediatric population, there has been a substantial reduction of vaccine serotype OM among children (3–6). However, despite the success of PCVs, OM disease burden remains substantial (7). Further, a greater proportion of pneumococcal OM cases due to non-PCV13 vaccine serotypes have been reported in the US in recent years (8, 9).

The emergence of non-PCV13 vaccine serotypes has led to the development and recommendation of higher-valency PCVs, including a 15-valent PCV (10) and a 20-valent PCV (11). Both vaccines have been approved for use among US children and indicated for prevention of invasive pneumococcal disease (IPD) due to the serotypes contained in the vaccines (10, 11). PCV20 is also indicated for prevention of OM caused by the original seven serotypes covered by PCV7 (11). Decisions pertaining to recent recommendations for pediatric PCV use have largely been informed by data from IPD whereas data from OM has been limited (12). While nasopharyngeal samples collected from children with OM have been used to approximate OM serotype distribution (13, 14), middle ear fluid (MEF) is still considered the gold standard specimen for detection and characterization of the causative OM agents.

The objective of this study was to assess the pneumococcal serotype distribution and antimicrobial susceptibility including multidrug resistance (MDR), and potential PCV coverage among isolates cultured from MEF obtained from children in the US.

## Materials and methods

### Pneumococcal isolate identification

The SENTRY Antimicrobial Surveillance Program was established in 1997 to monitor pathogens and changes in antimicrobial resistance patterns of organisms collected from patients with various infections (15). Every year, participating laboratories at medical centers in all 9 US Census Divisions identify requested surveillance pathogens by routine microbiologic methods and submit a subculture to the SENTRY program, along with basic demographic information about the case patient and limited information about the clinical setting where the case patient was treated. Pathogen confirmation is conducted by the central laboratory (JMI Laboratories, North Liberty, Iowa) by various techniques including colony morphology, biochemical algorithms, MALDI-TOF MS, PCR assays, and/or sequencing, as needed. From the SENTRY program collection of stored pathogens, *S. pneumoniae* isolates cultured from MEF samples from children 5 years of age or younger that were submitted from 35 participant laboratories during 2011–2021 were included in this study. Because tympanocentesis is not routinely performed among children with OM in the US and collection technique was not reported, we assumed that the MEF had been collected from a tympanocentesis performed on children with complicated OM (children with recurrent OM, children with a poor response to conventional therapy or children with a clinical relapse) or with a swab from otorrhea from a spontaneously perforated OM (16–18). We restricted serotyping to *S. pneumoniae* isolates collected from 2011 through 2021, after introduction of PCV13 into the US pediatric immunization program.

### Serotype determination

Serotypes were identified using *in silico* analysis based on Pneumococcal Capsular Typing methodology (PneumoCaT). Specifically, DNA from *S. pneumoniae* isolates was extracted using the KingFisher Cell and Tissue DNA kit on a KingFisher™ Flex Magnetic Particle Processor (Thermo Scientific) workstation. Total genomic DNA was used as input material for library construction and sequencing on a NextSeq 1000 Sequencer (Illumina, San Diego, California, USA) using NextSeq™1000/2000 P2 Reagents (300 cycles). The DNA libraries for the NextSeq1000 Sequencer were prepared using the Illumina DNA™ library construction protocol and index kit. Rigorous quality control metrics were applied to library construction including verification that the “Quality Score” (% ≥Q30) for the run was above 75%, the percentage of clusters passing filter was ≥60%, and the loading concentration was ≥95. Each raw data set was *de novo* assembled using SPAdes 3.11.1. PneumoCaT (v. 1.2.1) used a two-step approach to assign capsular type to *S. pneumoniae* genomic data. In the first step, if the reads matched >90% to one or more of the 92 serotypes for *S. pneumoniae* plus 2 additional subtypes/molecular types, a capsular type was assigned. If more than one loci matched, then in the second step, a variant-based approach that utilizes the capsular type variant database to distinguish serotypes within a subgroup/genogroup was applied to make a call on the serotype. If the coverage value against the reference sequence was ≤90%, then the serotype analysis reported “Failed” as its value. Average depth of coverage across the matching region of ≥30X was considered acceptable for PneumoCaT to analyze the data (19, 20). The nucleotide sequences used to assign a serotype were submitted to the National Center for Biotechnology Information (NCBI) and were assigned accession numbers SAMN41612263 - SAMN41612461.

### Susceptibility testing

Minimum Inhibitory Concentrations (MICs) were determined by the broth microdilution method using Clinical and Laboratory Standards Institute (CLSI) guidelines (2018) (21). Reference broth microdilution panels were manufactured at JMI Laboratories (2015–2021) or purchased from Thermo Fisher Scientific (2011–2014) (Cleveland, Ohio, USA) using freshly prepared drug stocks and stored at −80°C until use. Testing was performed in cation-adjusted Mueller-Hinton broth supplemented with 2.5% to 5.0% lysed horse blood (MHB-LHB)**.** Antimicrobial susceptibility testing was performed for amoxicillin-clavulanic acid, penicillin, ceftriaxone, clindamycin, erythromycin, trimethoprim/sulfamethoxazole (TMP/SMX), levofloxacin, and vancomycin. MIC results were interpreted as susceptible, intermediate, or resistant according to the CLSI recommendations (22). Non-meningitis breakpoints were used for penicillin and ceftriaxone. Non-susceptible isolates were defined as those isolates that were intermediate or resistant to a selected antimicrobial agent. Multidrug resistance (MDR) was defined as resistant to three or more classes of antimicrobials. The MIC_50_ was defined as the MIC of a given antimicrobial drug that inhibited growth of 50% of isolates, and MIC_90_ was defined as the MIC that inhibited growth of 90% of the isolates.

### Data analysis

Percentages of cases due to each serotype, non-susceptible to the selected antimicrobials, and covered by PCVs were calculated. PCV coverage was estimated for PCV7 (4, 6B, 9 V, 14, 18C, 19F, and 23F), PCV13 non-PCV7 (1, 3, 5, 6A, 7F, and 19A), PCV13 (PCV7 + PCV13 non-PCV7 serotypes), PCV15 (PCV13, 22F, and 33F), PCV15 non-PCV13 (22F and 33F), PCV20 non-PCV13 (8, 10A, 11A, 12F, 15B, 22F, and 33F), PCV20 (PCV13, PCV20 non-PCV13 serotypes), and non-PCV20 (serotypes not covered by PCV20). While serotypes 6C and 15C are not included in any PCV formulations, we also present PCV coverage estimates where these were grouped with PCVs that contained conjugates 6A or 15B due to potential prevention based on immunological or epidemiological evidence of cross-protection (23–25). Percentages were also stratified into two age groups (<2 years and 2–5 years) and two periods (to reflect potential changes associated with PCV13 introduction; Period 1 [P1: 2011–2016] and Period 2 [P2: 2017–2021]). Periods and age groups were compared for individual serotypes and PCV serotype groups by Chi-square test; *p*-values < 0.05 were considered significant. Non-susceptibility data were stratified by PCV group (PCV13, PCV20 non-PCV13, PCV15 non-PCV13, Non-PCV20). Analyses were performed using STATA (26).

## Results

### Study population

A total of 199 *S. pneumoniae* isolates from middle ear fluid among children 5 years of age or younger were collected by the SENTRY program from 2011 to 2021. All 199 *S. pneumoniae* were serotyped (by sequencing) and tested for antimicrobial susceptibility (Table 1). Of these, 113 isolates (56.8%) were from children <2 years of age. Most isolates were collected from children receiving care in the ear, nose, and throat (ENT; *n* = 89, 44.7%) or pediatrics (*n* = 61, 30.7%) departments. Approximately 60% of isolates came from male children, and about 50% were isolated between 2017 and 2021. While isolates were reported from all 9 US Census regions, most (57.3%) originated from the West North Central region.

**Table 1:** Characteristics of *S. pneumoniae* isolated from MEF samples.

Characteristics	*N* (%)
Total number of isolates	199
Age distribution	
0–<2 years	113 (56.8)
2–5 years	86 (43.3)
Male sex	119 (59.8)
Study years	
2011–2016	102 (51.3)
2017–2021	97 (48.7)
US Census Region	
New England	6 (3.0)
Mid-Atlantic	26 (13.1)
East North Central	31 (15.6)
West North Central	83 (41.7)
East South Central	6 (3.0)
West South Central	7 (3.5)
South Atlantic	22 (11.1)
Mountain	2 (1.0)
Pacific	16 (8.0)
Medical service location	
Ambulatory/outpatient	23 (11.6)
Emergency	13 (6.5)
Ear, nose, throat	89 (44.7)
Pediatrics	61 (30.7)
Surgery	6 (3.0)
Other^a^	7 (3.5)

^a^ Other service locations include Family Practice (*n* = 4), Hematology/Oncology (*n* = 1), Infectious Disease (*n* = 1), or not specified (*n* = 1).

### Serotype distribution

**Figure 1:**
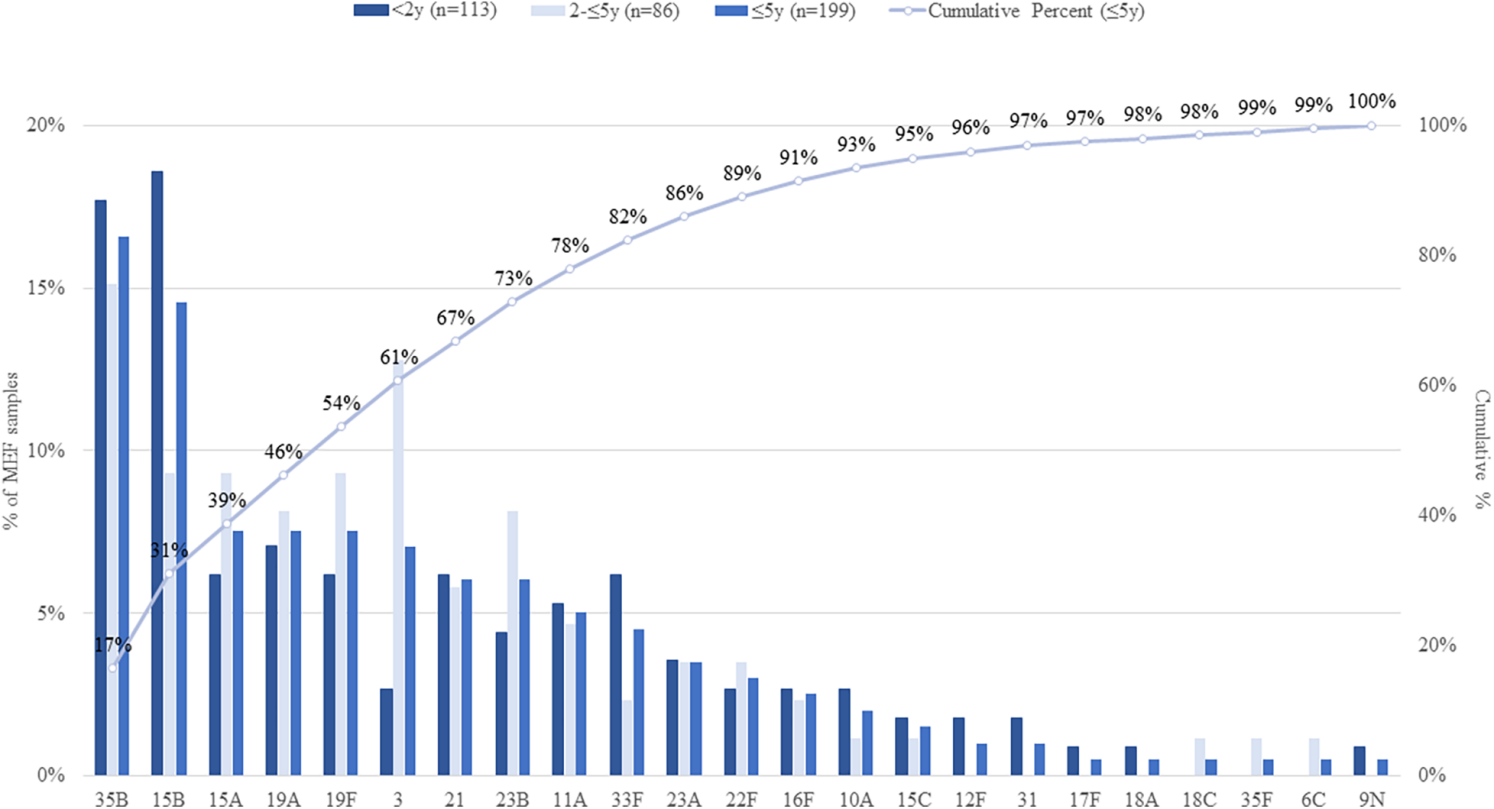
Distribution of *S. pneumoniae* serotypes isolated from MEF samples among children, by age group. Serotypes included in each PCV are: PCV7 serotypes = 4, 6B, 9V, 14, 18C, 19F, 23F; PCV13 serotypes = PCV7 serotypes and 1, 3, 5, 6A, 7F, 19A; PCV15 serotypes = PCV13 serotypes and 22F and 33F; PCV20 serotypes = PCV13 serotypes and 8, 10A, 11A, 12F, 15B, 22F, 33F.

**Figure 2:**
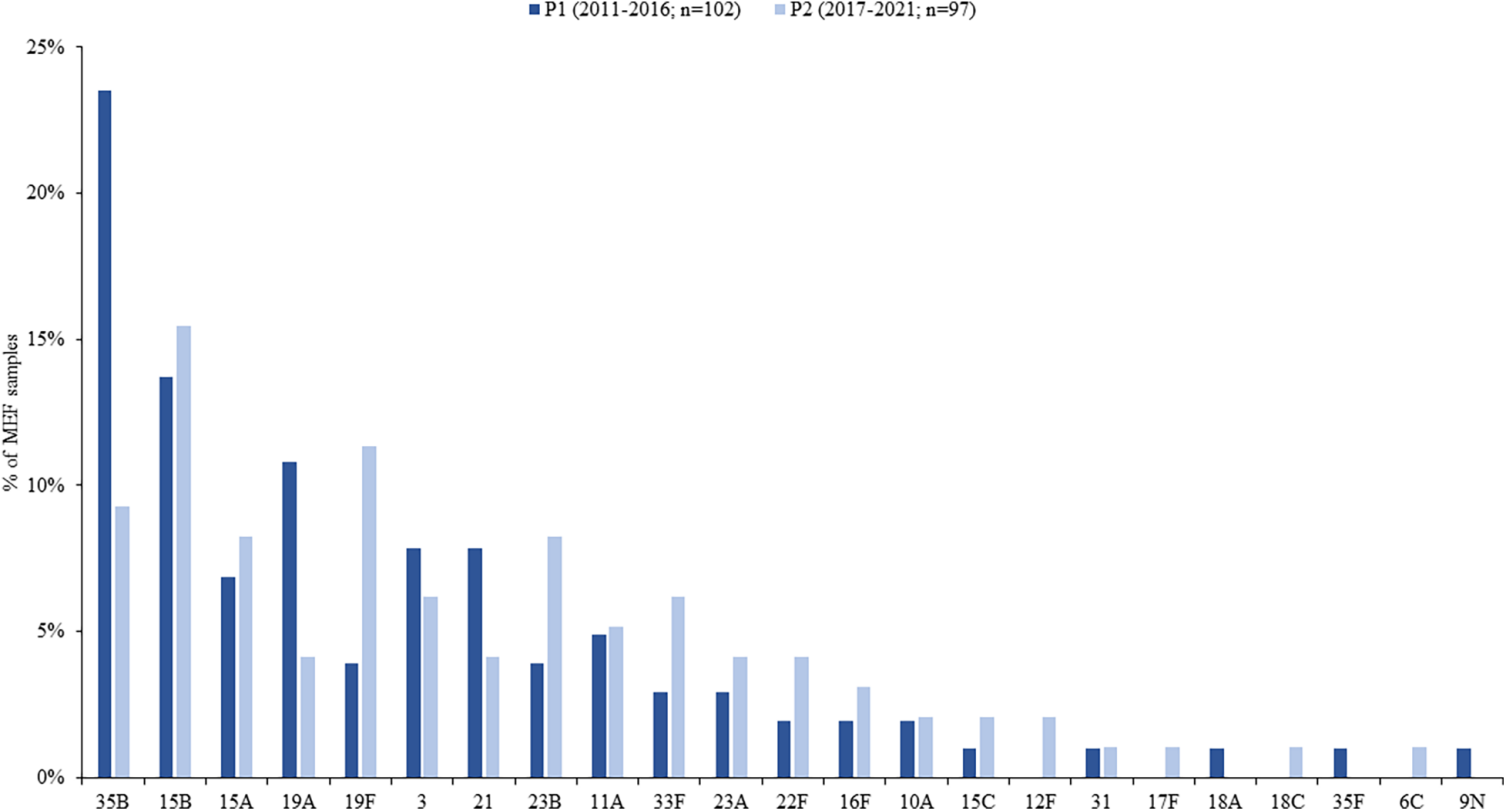
Distribution of *S. pneumoniae* serotypes isolated from MEF samples among children ≤5 years, by study period. Serotypes included in each PCV are: PCV7 serotypes = 4, 6B, 9V, 14, 18C, 19F, 23F; PCV13 serotypes = PCV7 serotypes and 1, 3, 5, 6A, 7F, 19A; PCV15 serotypes = PCV13 serotypes and 22F and 33F; PCV20 serotypes = PCV13 serotypes and 8, 10A, 11A, 12F, 15B, 22F, 33F.

Among the 199 *S. pneumoniae* isolates that were serotyped, twenty-three unique serotypes were identified. Roughly 60% of isolates were represented by 6 serotypes: 35B (16.6%), 15B (14.6%), 15A (7.5%), 19A (7.5%), 19F (7.5%), and 3 (7.0%) (Figure 1). Serotype 3 represented a higher percentage of total isolates among children 2–5 years of age than among those age <2 years (12.8% vs. 2.7%, respectively; *p*-value = 0.01), whereas serotype 15B represented a smaller percentage in older children (9.3% vs. 18.6%, respectively; *p*-value = 0.06). Comparing study periods, serotypes 35B and 15B represented the highest percentage of detected serotypes in P1 and P2, respectively (Figure 2 and Supplementary Table S1) whereas the percentage due to serotype 19A was higher in P1 than in P2 (10.8% vs. 4.1%, respectively; *p*-value = 0.08) and serotype 19F was higher in P2 than in P1 (11.3% vs. 3.9%, respectively; *p*-value = 0.048; Figure 2 and Supplementary Table S1). Serotype distribution by age group and study period are presented in Supplementary Table S1.

### Potential vaccine serotype coverage

In children aged ≤5 years, PCV13, PCV15, and PCV20 serotypes accounted for 23.1%, 30.2%, and 54.8% of *S. pneumoniae* isolates, respectively and 45.2% of cases were due to Non-PCV20 serotypes (Table 2). The PCV20 non-PCV13 serotypes and PCV15 non-PCV13 serotypes contributed 31.7% and 7.5% of cases, respectively (Table 2). Considering the age group, while PCV20 coverage was similar for children in both age groups, PCV20 non-PCV13 coverage was higher for children <2 years than in children 2–5 years of age (37.2% vs. 20.9%, respectively; *p*-value = 0.01) and this was due to a larger percentage of serotype 15B isolates detected in the younger age group. Overall, the percentage of serotypes included in PCV20 was higher in P2 than P1 (57.7% vs. 48.0%, respectively; *p*-value = 0.17) as was also true for PCV20 non-PCV13 serotypes (35.1% vs. 25.5%, respectively; *p*-value = 0.14) (Table 2). Serotype distribution by age group and study period are presented in Supplementary Table S2.

**Table 2:** Potential PCV serotype group coverage of *S. pneumoniae* isolated from MEF by age group (<2 years and 2–5 years) and study period (P1: 2011–2016 and P2: 2017–2021).

	Age group					Study period					Total	
	<2 years		2–5 years			P1 (2011–2016)		P2 (2017–2021)			≤5 years (2011–2021)	
Total isolates (*N*)	113		86			102		97			199	
PCV serotype groups	*N*	%	*N*	%	*p*-value	*N*	%	*N*	%	*p*-value	*N*	%
PCV20	60	53.1	45	52.3	0.91	49	48.0	56	57.7	0.17	105	52.8
PCV20 non-PCV13	42	37.2	18	20.9	0.01	26	25.5	34	35.1	0.14	60	30.2
PCV15	28	24.8	32	37.2	0.06	28	27.5	32	33.0	0.40	60	30.2
PCV15 non-PCV13	10	8.8	5	5.8	0.42	5	4.9	10	10.3	0.15	15	7.5
PCV13	18	15.9	27	31.4	0.01	23	22.5	22	22.7	0.98	45	22.6
PCV13 non-PCV7	11	9.7	18	20.9	0.02	19	18.6	10	10.3	0.10	29	14.6
PCV7	7	6.2	9	10.5	0.27	4	3.9	12	12.4	0.03	16	8.0
Non-PCV20	53	46.9	41	47.8	0.91	53	52.0	41	42.3	0.17	94	47.2
PCV serotype groups, including potentially preventable cross-reactive serotypes												
PCV20 plus 6C, 15C	62	54.9	47	54.7	0.98	50	49.0	59	60.8	0.09	109	54.8
PCV15 plus 6C	28	24.8	33	38.4	0.04	28	27.5	33	34.0	0.32	61	30.7
PCV13 plus 6C	18	15.9	28	32.6	0.01	23	22.5	23	23.7	0.85	46	23.1

P1 = Period 1; P2 = Period 2; PCV = pneumococcal conjugate vaccine; PCV7 serotypes = 4, 6B, 9V, 14, 18C, 19F, and 23F; PCV13 non-PCV7 serotypes = 1, 3, 5, 6A, 7F, and 19A; PCV13 serotypes = PCV7 and PCV13 non-PCV7 serotypes; PCV15 serotypes = PCV13 serotypes and serotypes 22F and 33F; PCV15 non-PCV13 serotypes = 22F and 33F; PCV20 serotypes = PCV13 serotypes and 8, 10A, 11A, 12F, 15B, 22F, and 33F; PCV20 non-PCV13 serotypes = 8, 10A, 11A, 12F, 15B, 22F, and 33F; Non-PCV20 serotypes = All remaining serotypes not covered by PCV20.

### Antimicrobial susceptibility

Non-susceptibility to penicillin, erythromycin, and TMP/SMX was common overall (45.2%, 49.8%, and 37.4%, respectively), but only 9.6% for amoxicillin-clavulanic acid (Table 3). Overall, 13.6% (*n* = 27) of isolates were MDR; most were PCV13 serotypes (*n* = 15) (Table 3). The proportions of non-susceptible isolates were similar across time periods for these antimicrobials, except for PCV13, for which proportions were lower in P2 than in P1 (Supplementary Table S3). Non-susceptibility to at least one antimicrobial was observed among 17 of the 23 serotypes identified. The majority of non-susceptibility was accounted for by serotypes 19A, 15A, 15B, 15C, 23A, 33F, and 35B (Table 4). More than 50% of serotype 19A isolates were non-susceptible to all antimicrobials tested, and 87% (*n* = 13) were MDR.

**Table 3:** Antimicrobial susceptibility of *S. pneumoniae* isolated from MEF obtained from children ≤5 years, 2011–2021.

Antimicrobial agent^a^		PCV serotype groups			
	Overall	PCV20 non-PCV13	PCV15 non-PCV13	PCV13	Non-PCV20
Total isolates	199	60	15	45	94
Amoxicillin-clavulanic acid					
Nonsusceptible, *n*/*N* isolates tested (%)	19/199 (9.6)	1/60 (1.7)	0/15 (0)	13/45 (28.9)	5/94 (5.3)
MIC_50_/MIC_90_ (range)	≤0.12/2 (≤0.06–8)	≤0.06/1 (≤0.06–4)	≤0.06/1 (≤0.06–1)	≤0.06/8 (≤0.06–8)	≤0.5/2 (≤0.06–4)
Penicillin (oral)					
Nonsusceptible, *n*/*N* isolates tested (%)	90/199 (45.2)	20/60 (33.3)	3/15 (20)	15/45 (33.3)	55/94 (58.5)
MIC_50_/MIC_90_ (range)	≤0.06/2 (≤0.06–8)	0.06/0.5 (≤0.06–4)	≤0.06/1 (≤0.06–1)	0.06/4 (≤0.06–8)	≤0.12/2 (≤0.06–2)
Ceftriaxone					
Nonsusceptible, *n*/*N* isolates tested (%)	9/199 (4.5)	1/60 (1.7)	0/15 (0)	8/45 (17.8)	0/94 (0)
MIC_50_/MIC_90_ (range)	≤0.06/1 (≤0.06–2)	≤0.06/≤0.5 (≤0.06–2)	≤0.06/≤0.5 (≤0.06–0.5)	≤0.06/2 (≤0.06–2)	≤0.12/1 (≤0.06–1)
Clindamycin					
Nonsusceptible, *n*/*N* isolates tested (%)	30/198 (15.2)	2/60 (3.3)	0/15 (0)	13/45 (28.9)	15/93 (16.1)
MIC_50_/MIC_90_ (range)	≤0.25/2 (≤0.125–2)	0.25/0.25 (≤0.125–2)	0.25/0.25 (≤0.125–2)	≤0.25/2 (≤0.12–2)	0.25/2 (≤0.125–2)
Erythromycin					
Nonsusceptible, *n*/*N* isolates tested (%)	99/199 (49.8)	36/60 (60)	11/15 (73.3)	16/45 (35.6)	47/94 (50)
MIC_50_/MIC_90_ (range)	≤0.12/16 (≤0.06–32)	2/8 (≤0.06–32)	≤0.06/4 (≤0.06–8)	0.06/16 (≤0.06–32)	0.5/16 (≤0.06–32)
TMP/SMX					
Nonsusceptible, *n*/*N* isolates tested (%)	74/198 (37.4)	29/60 (48.3)	9/15 (60)	18/44 (40.9)	27/94 (28.7)
MIC_50_/MIC_90_ (range)	≤0.5/≤9.5/4/76 (≤0.125/≤2.375–4/76)	0.5/9.5/4/76 (≤0.125/≤2.375–4/76)	1/19/4/76 (≤0.125/≤2.375–4/76)	≤0.5/≤9.5/4/76 (≤0.125/≤2.375–4/76)	0.5/9.5/4/76 (≤0.125/≤2.375–4/76)
MDR					
MDR, *n*/*N* isolates tested (%)	27/199 (13.6)	3/60 (5)	0/15 (0)	15/45 (33.3)	9/94 (9.6)

MDR = multidrug resistance, defined as resistance (R) to three or more classes of antimicrobials; MIC = minimum inhibitory concentration; PCV13 serotypes = 1, 3, 4, 5, 6A, 6B, 7F, 9V, 14, 18C, 19A, 19F, and 23F; PCV15 non-PCV13 serotypes = 22F and 33F; PCV20 non-PCV13 serotypes = 8, 10A, 11A, 12F, 15B, 22F, and 33F; Non-PCV20 serotypes = All remaining serotypes not covered by PCV20; SMX = sulfamethoxazole; TMP = trimethoprim.

^a^ Results for Levofloxacin and Vancomycin are not included because all isolates were susceptible to these antimicrobials. Nonsusceptible is defined as intermediate or resistant.

**Table 4:** Antimicrobial nonsusceptibility of *S. pneumoniae* serotypes isolated from MEF from children ≤5 years, 2011–2021.

	PCV serotype group																
	PCV20 non-PCV13^a^						PCV13				Non-PCV20						
Serotype	10A	11A	12F	15B	22F	33F	3	18C	19A	19F	15A	15C	21	23A	23B	31	35B
Total isolates per serotype	4	10	2	29	6	9	14	1	15	15	15	3	12	7	12	2	33
% nonsusceptible^b^, by antimicrobial^c^																	
Amoxicillin-clavulanic acid	0	0	0	3.4	0	0	0	0	86.7	0	0	0	0	0	0	0	15.2
Penicillin (oral)	0	20.0	0	51.7	0	33.3	7.1	0	93.3	0	86.7	66.7	0	71.4	50.0	0	87.9
Ceftriaxone	0	0	0	3.3	0	0	0	0	53.3	0	0	0	0	0	0	0	0
Clindamycin	0	0	0	6.9	0	0	7.1	0	80.0	0	80.0	0	0	28.6	0	0	3.0
Erythromycin	25.0	40.0	100	62.1	33.3	100	7.1	0	93.3	6.7	100	66.7	0	28.6	25.0	50.0	72.7
TMP/SMX	0	20.0	0	62.1	0	100	7.1	100	93.3	13.3	53.3	66.7	8.3	28.6	58.3	0	21.2
MDR	0	10.0	0	7	0	0	7.1	0	86.7	6.7	20.0	0	0	0	0	0	18.2

MDR = multidrug resistance; defined as resistance (R) to three or more classes of antimicrobials; PCV13 serotypes = 1, 3, 4, 5, 6A, 6B, 7F, 9V, 14, 18C, 19A, 19F, and 23F; PCV20 non-PCV13 serotypes = 8, 10A, 11A, 12F, 15B, 22F, and 33F; Non-PCV20 serotypes = All remaining serotypes not covered by PCV20; SMX = sulfamethoxazole; TMP = trimethoprim.

^a^ PCV15 non-PCV13 serotypes are 22F and 33F.

^b^ Nonsusceptible is defined as intermediate or resistant.

^c^ Results for Levofloxacin and Vancomycin are not included because all isolates were susceptible to these antimicrobials. No nonsusceptible isolates were identified for serotypes 6C, 9N, 16F, 17F, 18A, 35F.

## Discussion

In this study we assessed the serotype distribution, potential coverage by available pneumococcal vaccines, and antimicrobial susceptibility for pneumococcal isolates cultured from the MEF of US children with OM. Our results showed that more than half of *S. pneumoniae* isolates were serotypes covered by PCV20 (52.8%), 30.2% were covered by PCV15, and 22.6% were covered by PCV13. Serotypes 35B (16.6%) and 15B (14.6%) were the most common serotypes overall followed by serotypes 15A, 3, 19A, and 19F, each accounting for approximately 7% of cases. Together, these serotypes accounted for nearly two-thirds (61%) of pneumococcal isolates identified from MEF samples.

Other studies from US and European children that cultured and serotyped *S. pneumoniae* from MEF also reported that these serotypes were commonly identified. In these studies, and ours, the most common PCV13 serotypes collected from MEF were serotypes 3, 19A, and 19F (27–30). In the two US studies conducted during the PCV13 period among children with intact or perforated OM, serotypes 15A, 15B/C, 23B, and 35B also were among the most commonly identified (27, 29). In German and French children, serotypes 15B/C, 23B, and 35B, but not 15A as in our study, were among the most common serotypes detected in MEF. In addition, serotypes 10A, 11A, 16F, 24F were also common among French children only (28, 30).

Several explanations could account for these aggregate results. For PCV13 serotypes 3, 19A, and 19F, pre-licensure immunogenicity studies demonstrated that PCV13 elicited a robust immune response and functional antibody activity for each of these serotypes, particularly after the booster dose (31). Post-licensure studies reported that following PCV introduction population-based reductions (i.e., considering the combination of direct and indirect protection) declined significantly due to serotypes 19A and 19F and also for serotype 3, albeit not significantly (32), while PCV13 protected against each of these three serotypes in Israel (3). These data suggest that residual disease due to these serotypes could be attributed to lower effectiveness against carriage, including potentially against carriage acquisition, density, or duration of the immune response (33–36). For example, in our study, serotype 3 was more common among children 2–5 years (12.8%) than children <2 years of age (2.7%), which could reflect a shorter duration of immunity for serotype 3 (37). Alternatively, residual disease due to these serotypes could also be due to a lower amount of antibody present in the MEF or with a capsular phenotype of some serotypes (e.g., serotype 3) that facilitates progression to disease (38).

For the most common non-PCV13 serotypes identified in this study (15A, 15B, 15C, 23B, 35B), not only have they become increasingly common colonizers over subsequent eras of PCV use, but they have also been frequently associated with antimicrobial nonsusceptibility, especially serotypes 15A and 35B (9, 27, 39). Serotype 35B has also become both a more common invasive serotype and acquired multiclass non-susceptibility among US children with IPD (40). The emergence of these non-susceptible strains likely results from selection pressure created by exposure to antimicrobials (41). Strains possessing both non-susceptibility and virulence genes may become more common causes of systemic and mucosal disease as was observed among certain non-vaccine serotypes after PCV7 introduction (40, 42).

Overall, a substantial proportion of isolates in each PCV serotype group was non-susceptible to at least one antimicrobial. While non-susceptibility was seen for at least one isolate for most of the 23 serotypes identified, most non-susceptible isolates were accounted for by 7 serotypes: 19A, 15A, 15B, 15C, 23A, 33F, and 35B, most of which were also reported by other studies (9, 40). We also observed a decrease in the proportion of non-susceptible and MDR PCV13 serotypes over the study time periods, primarily driven by the decrease of serotype 19A, as has been reported previously (40, 43). PCV15 includes serotypes 19A and 33F and PCV20 also includes serotypes 15B and possibly 15C through cross protection (24), with the latter potentially covering 39% of penicillin non-susceptible isolates. Serotypes 15A, 23A, and 35B are not currently covered by any higher valency PCVs, and while disease incidence for these serotypes has not increased, non-susceptibility to antimicrobials among these serotypes is common, which makes them important candidates to include in future higher valency PCVs (40). Importantly, serotype inclusion and PCV coverage estimates may not linearly translate to protection due to immune interference and the possible need for higher levels of antibody to prevent mucosal disease (36).

Our study had limitations. Because the source of many *S. pneumoniae* isolates was presumably from children with spontaneously perforated OM, the serotype distribution of these cases may not be representative of all OM cases. Specifically, the majority of children were seen in the ENT or pediatrics departments which could be indicative of prior antibiotic treatment failure. Clinical data such as previous use of antimicrobials and vaccine history were not available, and therefore, it was not possible to associate *S. pneumoniae* isolation with therapeutic failure or vaccine breakthrough infection. Also, the SENTRY program is a laboratory-based surveillance system designed to observe distribution of pathogens and antimicrobial resistance patterns for any given infection based on a prespecified target number of pathogens per year. Therefore, the true prevalence of *S. pneumoniae* serotypes cannot be ascertained with these sampling criteria. However, this study demonstrates that antimicrobial resistance surveillance systems can be leveraged to understand serotype distribution of pneumococcal isolates from MEF samples.

Our study showed that certain PCV13 serotypes – 3, 19A, and 19F – are frequently isolated from MEF of children even in the context of a mature pediatric PCV13 program, emphasizing the need for continued monitoring following the introduction of higher valency vaccines such as PCV15 and PCV20. Furthermore, among the pneumococcal isolates in this study, an important fraction were attributed to serotypes beyond those in PCV13 that are covered by higher valency PCVs. Among the additional serotypes in PCV20 beyond PCV13, several were associated with antimicrobial non-susceptibility and MDR. Therefore, including PCV20 in existing pediatric national immunization programs may further reduce the frequency of overall and antimicrobial non-susceptible *S. pneumoniae*. With the introduction of higher valency PCVs, further studies are needed to monitor the changes in pneumococcal epidemiology in children with OM in the long term. Future PCVs should consider including non-PCV20 serotypes, particularly 15A, 23A, and 35B, to further reduce the burden of OM among children.

## Data Availability

The raw data supporting the conclusions of this article will be made available by the authors, without undue reservation.
