# Supplementary material for: Characterization of Streptococcus pneumoniae isolates obtained from the middle ear fluid of US children, 2011–2021
Source: Front Pediatr. 2024 Jul 15;12:1383748. doi: 10.3389/fped.2024.1383748 (PMC11284096; doi:10.3389/fped.2024.1383748)
Supplement: Supplementary file 1 [file Datasheet1.pdf]

Supplementary Tables

Supplementary Table 1: Serotype distribution of *S. pneumoniae* isolated from MEF by age group (<2 years, 2-5 years, and ≤5 years) and study period (P1: 2011-2016 and P2: 2017-2021)

	**Age Group**																	
	**<2 years**						**2-5 years**						**≤5 years**					
**Study Period**	**P1 (2011-2016)**		**P2 (2017-2021)**		**Total (2011-2021)**		**P1 (2011-2016)**		**P2 (2017-2021)**		**Total (2011-2021)**		**P1 (2011-2016)**		**P2 (2017-2021)**		**Total (2011-2021)**	
**Total isolates**	**57**		**56**		**113**		**45**		**41**		**86**		**102**		**97**		**199**	
**Serotype**	**N**	**%**	**N**	**%**	**N**	**%**	**N**	**%**	**N**	**%**	**N**	**%**	**N**	**%**	**N**	**%**	**N**	**%**
**PCV20 non-PCV13[a]**	**18**	**31.6**	**24**	**42.9**	**42**	**37.2**	**8**	**17.8**	**10**	**24.4**	**18**	**20.9**	**26**	**25.5**	**34**	**35.1**	**60**	**30.2**
10A	2	3.5	1	1.8	3	2.7	0	0	1	2.4	1	1.2	2	2.0	2	2.1	4	2.0
11A	3	5.3	3	5.4	6	5.3	2	4.4	2	4.9	4	4.7	5	4.9	5	5.2	10	5.0
12F	0	0	2	3.6	2	1.8	0	0	0	0	0	0	0	0.0	2	2.1	2	1.0
15B	10	17.5	11	19.6	21	18.6	4	8.9	4	9.8	8	9.3	14	13.7	15	15.5	29	14.6
22F	1	1.8	2	3.6	3	2.7	1	2.2	2	4.9	3	3.5	2	2.0	4	4.1	6	3.0
33F	2	3.5	5	8.9	7	6.2	1	2.2	1	2.4	2	2.3	3	2.9	6	6.2	9	4.5
**PCV13**	**7**	**12.3**	**11**	**19.6**	**18**	**15.9**	**16**	**35.6**	**11**	**26.8**	**27**	**31.4**	**23**	**22.5**	**22**	**23.7**	**45**	**22.6**
3	0	0	3	5.4	3	2.7	8	17.8	3	7.3	11	12.8	8	7.8	6	6.2	14	7.0
18C	0	0	0	0	0	0	0	0	1	2.4	1	1.2	0	0.0	1	1.0	1	0.5
19A	6	10.5	2	3.6	8	7.1	5	11.1	2	4.9	7	8.1	11	10.8	4	4.1	15	7.5
19F	1	1.8	6	10.7*	7	6.2	3	6.7	5	12.2	8	9.3	4	3.9	11	11.3	15	7.5*
**Non-PCV20**	**32**	**56.1**	**21**	**37.57***	**53**	**46.9**	**21**	**46.7**	**20**	**48.8**	**41**	**47.8**	**53**	**52.0**	**41**	**42.3**	**94**	**47.2**
6C	0	0	0	0	0	0	0	0	1	2.4	1	1.2	0	0.0	1	1.0	1	0.5
9N	1	1.8	0	0	1	0.9	0	0	0	0	0	0	1	1.0	0	0.0	1	0.5
15A	4	7	3	5.4	7	6.2	3	6.7	5	12.2	8	9.3	7	6.9	8	8.2	15	7.5
15C	1	1.8	1	1.8	2	1.8	0	0	1	2.4	1	1.2	1	1.0	2	2.1	3	1.5

16F	1	1.8	2	3.6	3	2.7	1	2.2	1	2.4	2	2.3	2	2.0	3	3.1	5	2.5
17F	0	0	1	1.8	1	0.9	0	0	0	0	0	0	0	0.0	1	1.0	1	0.5
18A	1	1.8	0	0	1	0.9	0	0	0	0	0	0	1	1.0	0	0.0	1	0.5
21	5	8.8	2	3.6	7	6.2	3	6.7	2	4.9	5	5.8	8	7.8	4	4.1	12	6.0
23A	1	1.8	3	5.4	4	3.5	2	4.4	1	2.4	3	3.5	3	2.9	4	4.1	7	3.5
23B	1	1.8	4	7.1	5	4.4	3	6.7	4	9.8	7	8.1	4	3.9	8	8.2	12	6.0
31	1	1.8	1	1.8	2	1.8	0	0	0	0	0	0	1	1.0	1	1.0	2	1.0
35B	16	28.1	4	7.1	20	17.7	8	17.8	5	12.2	13	15.1	24	23.5	9	9.3	33	16.6*
35F	0	0	0	0	0	0	1	2.2	0	0	1	1.2	1	1.0	0	0.0	1	0.5

Abbreviations: P1 = Period 1; P2 = Period 2; PCV = pneumococcal conjugate vaccine; PCV13 serotypes = 1, 3, 4, 5, 6A, 6B, 7F, 9V, 14, 18C, 19A, 19F, and 23F; PCV20 non-PCV13 serotypes = 8, 10A, 11A, 12F, 15B, 22F, and 33F; Non-PCV20 = All remaining serotypes not covered by PCV20; *p-value<0.05

a. PCV15 non-PCV13 serotypes are 22F and 33F.

Supplementary Table 2. Potential pneumococcal vaccine serotype coverage by age group (<2 years and 2-5 years) and study period (P1: 2011-2016 and P2: 2017-2021)

	Age Group							
	<2 years				2-5 years			
**Age Group**	**P1 (2011-2016)**		**P2 (2017-2021)**		**P1 (2011-2016)**		**P2 (2017-2021)**	
**Total cases**	**57**		**56**		**45**		**41**	
**PCV serotype groups**	**N**	**%**	**N**	**%**	**N**	**%**	**N**	**%**
PCV20	25	43.9	35	62.5	24	53.3	21	51.2
PCV20 non-PCV13	18	31.6	24	42.9	8	17.8	10	24.4
PCV15	10	17.5	18	32.1	18	40.0	14	34.1
PCV15 non-PCV13	3	5.3	7	12.5	2	4.4	3	7.3
PCV13	7	12.3	11	19.6	16	35.6	11	26.8
PCV13 non-PCV7	6	10.5	5	8.9	13	28.9	5	12.2
PCV7	1	1.8	6	10.7*	3	6.7	6	14.6
Non-PCV20	32	56.1	21	37.5*	21	46.7	20	48.8
**PCV serotype groups, including potentially preventable cross-reactive serotypes**								
PCV20 plus 6C, 15C	26	45.6	36	64.3	24	53.3	23	56.1
PCV15 plus 6C	10	17.5	18	32.1	18	40.0	15	36.6
PCV13 plus 6C	7	12.3	11	19.6	16	35.6	12	29.3

Abbreviations: P1 = Period 1; P2 = Period 2; PCV = pneumococcal conjugate vaccine; PCV7 serotypes = 4, 6B, 9V, 14, 18C, 19F, and 23F; PCV13 non-PCV7 serotypes = 1, 3, 5, 6A, 7F, and 19A; PCV13 serotypes = PCV7 serotypes and PCV13 non-PCV7 serotypes; PCV15 serotypes = PCV13 serotypes and 22F and 33F; PCV15 non-PCV13 serotypes = 22F and 33F; PCV20 serotypes = PCV13 serotypes and 8, 10A, 11A, 12F, 15B, 22F, and 33F; PCV20 non-PCV13 serotypes = 8, 10A, 11A, 12F, 15B, 22F, and 33F; Non-PCV20 = All remaining serotypes not covered by PCV20; *p-value<0.05

Supplementary Table 3. Antimicrobial nonsusceptibility of *S. pneumoniae* isolated from MEF by study period among children ≤5 years (P1: 2011-2016 and P2: 2017-2021)

	**PCV Serotype Group**									
	**Overall**		**PCV20 non-PCV13**		**PCV15 non-PCV13**		**PCV13**		**Non-PCV20**	
**Study Period**	**P1 (2011-2016)**	**P2 (2017-2021)**	**P1 (2011-2016)**	**P2 (2017-2021)**	**P1 (2011-2016)**	**P2 (2017-2021)**	**P1 (2011-2016)**	**P2 (2017-2021)**	**P1 (2011-2016)**	**P2 (2017-2021)**
**Total isolates**	102	97	26	34	5	10	23	22	53	41
**Antimicrobial[a]**										
**Amoxicillin-clavulanic acid**										
Nonsusceptible[b], n/N isolates tested (%)	15/102 (14.7)	4/97 (4.2)	1/26 (3.9)	0/34 (0)	0/5 (0)	0/10 (0)	10/23 (43.5)	3/22 (13.6)	4/53 (7.5)	1/41 (2.4)
MIC_{50}/MIC_{90} (range)	≤1/4 (≤0.06-8)	≤0.06/2 (≤0.06-4)	≤1/1 (≤0.06-4)	≤0.06/0.5 (≤0.06-1)	≤1/1 (≤0.06-1)	≤0.06/1 (≤0.06-1)	≤1/8 (≤0.06-8)	≤0.06/4 (≤0.06-4)	≤1/2 (≤0.06-4)	≤0.06/2 (≤0.06-4)
**Penicillin (oral)**										
Nonsusceptible, n/N isolates tested (%)	55/102 (53.9)	35/97 (36.1)	10/26 (38.5)	10/34 (29.4)	1/5 (20)	2/10 (20)	12/23 (52.2)	3/22 (13.6)	33/53 (62.3)	22/41 (53.7)
MIC_{50}/MIC_{90} (range)	≤0.125/4 (≤0.06-8)	≤0.06/1 (≤0.06-4)	≤0.125/2 (≤0.06-4)	≤0.06/0.25 (≤0.06-1)	≤0.06/1 (≤0.06-1)	≤0.06/1 (≤0.06-1)	≤0.125/4 (≤0.06-8)	≤0.06/4 (≤0.06-4)	0.25/2 (≤0.06-2)	≤0.125/1 (≤0.06-2)
**Ceftriaxone**										
Nonsusceptible, n/ N isolates tested (%)	7/102 (6.9)	2/97 (2.1)	1/26 (3.9)	0/34 (0)	0/5 (0)	0/10 (0)	6/23 (26.1)	2/22 (9.1)	0/53 (0)	0/41 (0)

MIC_{50}/MIC_{90} (range)	≤0.125/1 (≤0.06-2)	≤0.06/0.5 (≤0.06-2)	≤0.06/1 (≤0.06-2)	≤0.06/0.25 (≤0.06-0.5)	≤0.06/0.5 (≤0.06-0.5)	≤0.06/≤0.5 (≤0.06-0.5)	≤0.06/2 (≤0.06-2)	≤0.06/1 (≤0.06-2)	0.25/1 (≤0.06-1)	0.06/0.5 (≤0.06-1)
**Clindamycin**										
Nonsusceptible, n/ N isolates tested (%)	20/101 (19.8)	10/97 (10.3)	2/26 (7.7)	0/34 (0)	0/5 (0)	0/10 (0)	11/23 (47.8)	2/22 (9.1)	7/52 (13.5)	8/41 (19.5)
MIC_{50}/MIC_{90} (range)	≤0.125/2 (≤0.125-2)	0.25/2 (0.25-2)	≤0.25/≤0.25 (≤0.125-2)	0.25/0.25 (0.25-0.25)	≤0.25/0.25 (≤0.125-0.25)	0.25/0.25 (0.25-2)	≤0.25/2 (≤0.125-2)	0.25/0.25 (0.25-2)	≤0.25/1 (≤0.125-2)	0.25/2 (0.25-2)
**Erythromycin**										
Nonsusceptible, n/ N isolates tested (%)	57/102 (55.9)	42/97 (43.3)	16/26 (61.5)	20/34 (58.8)	4/5 (80)	7/10 (70)	12/23 (52.2)	4/22 (18.2)	29/53 (54.7)	18/41 (43.9)
MIC_{50}/MIC_{90} (range)	2/16 (≤0.06-32)	0.06/16 (≤0.06-16)	2/16 (≤0.06-32)	≤2/8 (≤0.06-16)	4/8 (≤0.125-8)	4/8 (≤0.06-8)	2/16 (≤0.06-32)	≤0.06/16 (≤0.06-16)	2/16 (≤0.06-32)	≤0.06/16 (≤0.06-16)
**TMP/SMX**										
Nonsusceptible, n/ N isolates tested (%)	38/102 (37.3)	36/96 (37.5)	13/26 (50)	16/34 (47.1)	3/5 (60)	6/10 (60)	12/23 (52.2)	6/21 (28.6)	13/53 (24.5)	14/41 (34.2)
MIC_{50}/MIC_{90} (range)	≤0.5/≤9.5 / 4/76 (≤0.125/≤2.375 – 4/76)	0.25/4.75/ 4/76 (≤0.125/≤2.375-4/76)	≤2/≤38 / 4/76 (≤0.125/≤2.375 – 4/76)	0.25/4.75/ 2/38 (≤0.125/≤2.375-4/76)	2/38/ 4/76 (≤0.5/≤9.5 – 4/76)	1/19/ 3/57 (≤0.125/≤2.375-4/76)	4/76 / 4/76 (≤0.125/≤2.375 – 4/76)	0.25/4.75/ 4/76 (≤0.125/≤2.375-4/76)	≤0.5/≤9.5 / 4/76 (≤0.125/≤2.375 – 4/76)	0.25/4.75/ 2/38 (≤0.125/≤2.375-4/76)
**MDR**										
MDR, n/ N isolates tested (%)	20/102 (19.6)	7/97 (7.2)	3/26 (11.5)	0/34 (0)	0/5 (0)	0/10 (0)	11/23 (47.8)	4/22 (18.2)	6/53 (11.3)	3/41 (7.3)

Abbreviations: MDR = multidrug resistance, defined as resistance (R) to three or more classes of antimicrobials. MIC = minimum inhibitory concentration; P1 = Period 1; P2 = Period 2; PCV13 serotypes = 1, 3, 4, 5, 6A, 6B, 7F, 9V, 14, 18C, 19A, 19F, and 23F; PCV15 non-PCV13 serotypes = 22F and 33F; PCV20 non-PCV13 serotypes = 8, 10A, 11A, 12F, 15B, 22F, and 33F; Non-PCV20 serotypes = All remaining serotypes not covered by PCV20; SMX = sulfamethoxazole; TMP = trimethoprim;

a. Results for Levofloxacin and Vancomycin are not included because all isolates were susceptible to these antimicrobials.

b. Nonsusceptible is defined as intermediate or resistant.
